# Lipopolysaccharide and Tumor Necrosis Factor Alpha Inhibit Interferon Signaling in Hepatocytes by Increasing Ubiquitin-Like Protease 18 (USP18) Expression

**DOI:** 10.1128/JVI.02557-15

**Published:** 2016-05-27

**Authors:** Sonya A. MacParland, Xue-Zhong Ma, Limin Chen, Ramzi Khattar, Vera Cherepanov, Markus Selzner, Jordan J. Feld, Nazia Selzner, Ian D. McGilvray

**Affiliations:** a Multi-Organ Transplant Program, University Health Network, University of Toronto, Toronto, Ontario, Canada; b Institute of Blood Transfusion, Chinese Academy of Medical Science, Chengdu, China; c Toronto Centre for Liver Disease, McLaughlin-Rotman Centre for Global Health, University of Toronto, Toronto, Ontario, Canada; d Toronto Western Hospital, Toronto, Ontario, Canada

## Abstract

Inflammation may be maladaptive to the control of viral infection when it impairs interferon (IFN) responses, enhancing viral replication and spread. Dysregulated immunity as a result of inappropriate innate inflammatory responses is a hallmark of chronic viral infections such as, hepatitis B virus and hepatitis C virus (HCV). Previous studies from our laboratory have shown that expression of an IFN-stimulated gene (ISG), ubiquitin-like protease (USP)18 is upregulated in chronic HCV infection, leading to impaired hepatocyte responses to IFN-α. We examined the ability of inflammatory stimuli, including tumor necrosis factor alpha (TNF-α), lipopolysaccharide (LPS), interleukin-6 (IL-6) and IL-10 to upregulate hepatocyte USP18 expression and blunt the IFN-α response. Human hepatoma cells and primary murine hepatocytes were treated with TNF-α/LPS/IL-6/IL-10 and USP18, phosphorylated (p)-STAT1 and myxovirus (influenza virus) resistance 1 (Mx1) expression was determined. Treatment of Huh7.5 cells and primary murine hepatocytes with LPS and TNF-α, but not IL-6 or IL-10, led to upregulated USP18 expression and induced an IFN-α refractory state, which was reversed by USP18 knockdown. Liver inflammation was induced in vivo using a murine model of hepatic ischemia/reperfusion injury. Hepatic ischemia/reperfusion injury led to an induction of USP18 expression in liver tissue and promotion of lymphocytic choriomeningitis replication. These data demonstrate that certain inflammatory stimuli (TNF-α and LPS) but not others (IL-6 and IL-10) target USP18 expression and thus inhibit IFN signaling. These findings represent a new paradigm for how inflammation alters hepatic innate immune responses, with USP18 representing a potential target for intervention in various inflammatory states.

**IMPORTANCE** Inflammation may prevent the control of viral infection when it impairs the innate immune response, enhancing viral replication and spread. Blunted immunity as a result of inappropriate innate inflammatory responses is a common characteristic of chronic viral infections. Previous studies have shown that expression of certain interferon-stimulated genes is upregulated in chronic HCV infection, leading to impaired hepatocyte responses. In this study, we show that multiple inflammatory stimuli can modulate interferon stimulated gene expression and thus inhibit hepatocyte interferon signaling via USP18 induction. These findings represent a new paradigm for how inflammation alters hepatic innate immune responses, with the induction of USP18 representing a potential target for intervention in various inflammatory states.

## INTRODUCTION

Interferon (IFN) is a key endogenous mediator of viral clearance by the innate immune response. One excellent example of this is hepatitis C virus (HCV) infection of the liver (1). Interferon signaling drives the expression of multiple interferon-stimulated genes (ISGs), which mediate viral clearance. ISGs, however, can also be induced by other factors, including inflammatory stimuli such as tumor necrosis factor alpha (TNF-α) (2). HCV infection, which induces chronic inflammation of the liver, is associated with high serum TNF-α (3, 4). Interestingly, anti-TNF-α treatment has been shown to lead to an improved virologic response to IFN-α/ribavirin antiviral therapy for HCV (5), while other data show safety of combined treatment but no effect on HCV viral loads of HCV treatment-naive individuals (6). The purpose of the present study is to define an important causal link between inflammation and the host hepatic innate immune response, with broad viral relevance.

Our recent work in the host innate immune response to chronic HCV infection suggested an association between hepatocytes and the liver resident immune cells that drive liver inflammation (7–9). There is a dichotomous response to chronic HCV infection in the liver, and this response predicts who will and who will not respond to exogenous IFN-α treatment. In patients that do not respond to therapy with IFN/ribavirin, hepatocytes have strong preactivation of the IFN-response, with high expression of a subset of ISGs (7–9). In patients exhibiting a sustained virologic response on therapy with IFN/ribavirin, tissue macrophages show a high expression of ISGs (7, 9). The expression of ISGs in macrophages or hepatocytes is more predictive of treatment outcome than IL28B polymorphisms (7); this finding suggests a link between hepatic inflammatory cell activation and viral clearance.

Liver Kupffer cell inflammatory responses are induced by and play a role in the control and clearance of multiple viral infections of the liver (10, 11). Although the mechanisms underlying these patterns are undoubtedly complex, the association between liver macrophages and hepatocytes raises the question of how the hepatocytes react to the cytokines produced by the macrophages (and other inflammatory cells). If ISG expression in hepatocytes reflects a response to inflammatory stimuli, then how the liver responds to a viral infection will be modulated by the inflammatory milieu.

In HCV infection, we have found that the ISG15/USP18 pathway is an important regulatory pathway. Both ISG15 and USP18 are ISGs that are strongly upregulated in the livers of IFN treatment-resistant patients (7–9). ISG15 is a ubiquitin-like protein that is strongly upregulated by IFN and conjugates to multiple cellular proteins (12, 13). USP18 is an ISG15-specific protease that is also upregulated by IFN-α (12, 13). Both ISG15 and USP18 have been suggested to blunt type 1 IFN signaling (12, 14–16). In addition to its ability to remove ISG15 from its conjugated proteins, USP18 has been shown to bind to the type 1 IFN receptor and blunt IFN signaling (15). Upregulation of USP18 may represent a negative-feedback loop counteracting the effects of type 1 IFN. Furthermore, knockdown of USP18 increases both ISG induction and anti-HCV activity of IFN-α (14), and data have shown that IFN-α treatment given to mice in vivo increases hepatic USP18 and blunts the effect of a subsequent dose of IFN-α (12). The mechanisms controlling USP18 expression in the liver are poorly understood but will have relevance to understanding innate immune mechanisms in chronic viral infection.

Although the majority of work on USP18 has centered on its upregulation by type 1 IFN, interferon stimulated genes have multiple upregulatory stimuli other than IFN-α (2, 17). For example, inflammatory stimuli, e.g., endotoxin and lipopolysaccharide (LPS), have been shown to upregulate USP18 in peritoneal exudate macrophages (18). If similar stimuli lead to increased USP18 expression in hepatocytes, then this pathway could represent a novel link between inflammatory and innate immune responses in the liver. The present study is based on the hypothesis that liver inflammation will directly impact hepatocyte expression of USP18 and therefore will impact IFN signaling. This link will have relevance to multiple diseases, since inflammatory stimuli such as TNF-α and LPS have been shown to play roles in many liver diseases, including viral hepatitis (19–21). The link may also help to explain our observations in chronic HCV infection, since chronic HCV infection is characterized by increased serum TNF-α (3, 4), increased hepatocyte USP18, and impaired IFN responsiveness (8). Quite apart from HCV, the importance of the link between hepatic inflammation and innate immune response lies in the downstream effects of the impairment of innate immunity. We speculate that by blocking IFN-α signaling, USP18 expression may lead to an enhanced susceptibility to infection with interferon-sensitive viruses and enhanced viral proliferation. Support for this notion is found in a mouse study in which expression of USP18 in macrophages led to lower IFN responsiveness, leading to locally restricted replication of VSV (22).

In the present study, treatment of hepatic cells with LPS and TNF-α, but not IL-6 or IL-10, led to upregulated USP18 expression in hepatocytes. The enhanced USP18 expression was associated with decreased IFN-α-stimulated expression of p-STAT1 and ISGs, a phenomenon reversed by USP18 knockdown. As an *in vivo* correlate of our *in vitro* findings, experimentally induced hepatic ischemia/reperfusion injury induced USP18 mRNA expression in liver and enhanced lymphocytic choriomeningitis (LCMV) replication, an effect not seen in USP18^−/−^ mice. These data demonstrate that certain inflammatory stimuli (TNF-α and LPS), as well as ischemic injury, but not other cytokines (IL-6 and IL-10) can lead to enhanced hepatocyte USP18 expression and thereby inhibit IFN signaling. These findings lend new knowledge to our understanding of how inflammation can modulate hepatic innate immune responses, with USP18 representing a potential target for intervention to reverse any proviral effect of inflammation.

## MATERIALS AND METHODS

### Cell lines, culture reagents, and inflammatory stimuli.

Huh7.5 cells, a human hepatoma cell line, were provided by Charles Rice (Rockefeller University, NY) (23). Cells were maintained at 37°C in Dulbecco modified Eagle medium (DMEM) supplemented 10% fetal bovine serum (FBS; HyClone, Logan, UT), 100 U of penicillin, and 100 mg of streptomycin (Invitrogen, Carlsbad, CA). Inflammatory stimuli included TNF-α at 20 ng/ml (24) (R&D Systems, Minneapolis, MN), LPS from Escherichia coli at 100 ng/ml (25) (Sigma, Oakville, Ontario, Canada), IL-6 at 100 ng/ml (26) (Akron Biotech, Boca Raton, FL), IL-10 at 10 ng/ml (26) (Akron Biotech), and human IFN-α at 100 IU/ml (27) (PBL Interferon Source, Piscataway, NJ).

### Animals.

USP18^+/+^ and USP18^−/−^ C57BL/6 mice between 6 and 8 weeks were used for experiments. USP18^−/−^ mice were kindly provided by Dong Er Zhang (http://jaxmice.jax.org/strain/007225.html) (Scripps Research Institute, CA). Animals were housed in specific-pathogen-free conditions and treated according to the guidelines of the Canadian Council on Animal Care, and all procedures were approved by the University Health Network Animal Care Committee.

### Preparation of primary murine hepatocytes.

Hepatocytes from USP18^+/+^ and USP18^−/−^ mice were isolated as previously described (27). Briefly, mice were anesthetized by a 100-mg/kg intraperitoneal injection of pentobarbital. The portal vein was cannulated with a 21-gauge needle. The liver was flushed via the portal vena cava with liver perfusion medium (2 mM EGTA in Hanks balanced salt solution [Invitrogen] without Ca^2+^ or Mg^2+^) at 37°C at a rate of 7 ml/min using an infusion pump for 5 min. The liver was then perfused with liver digestion medium (0.02% collagenase IV; Sigma) at the same pressure. The liver was then placed in DMEM and 20 nM insulin (Sigma), minced and filtered through a 100-μm-pore size nylon mesh (BD Biosciences), and centrifuged for 3 min at 50 × *g* at room temperature. The cells were washed three times, and viability was confirmed on a Vi-Cell XR 2.03 (Beckman Coulter, Fullerton, CA) and was routinely determined to be 80 to 90%. Hepatocytes were plated at 10^6^ cells/ml in DMEM with 10% FBS on a six-well plate for 4 h, and the medium was replaced with serum-free DMEM supplemented with 20 nM insulin (Sigma), 5 μg of transferrin (Sigma)/ml, and 100 nM dexamethasone (Sigma).

### Western blot analysis.

Huh7.5 cells and primary murine hepatocytes were washed with phosphate-buffered saline and lysed with 100 μl of cell lysis buffer (BD Biosciences, San Jose, CA). The protein concentration was determined by a Bradford assay, and a 20-μg cell lysate was analyzed by SDS-PAGE. Proteins were probed with mouse anti-human USP18 polyclonal antibody (Abnova, Taipei, Taiwan), rabbit polyclonal anti-ISG15 (Santa Cruz Biotechnology, Santa Cruz, CA), rabbit polyclonal anti-phosphorylated-STAT1 (p-STAT-1; Cell Signaling Technology, Boston, MA), rabbit polyclonal anti-STAT-1 (Santa Cruz Biotechnology), goat polyclonal anti-UBE1 antibody (Santa Cruz Biotechnology), or mouse monoclonal anti-actin (Sigma) antibodies, followed by anti-mouse or anti-rabbit IgG conjugated to horseradish peroxidase (Calbiochem, Billerica, MA). An enhanced chemiluminescence detection kit (Amersham Pharmacia Biotech, Uppsala, Sweden) was used to determine the levels of protein expression.

### Flow cytometric quantification of USP18 expression in Huh7.5 cells.

USP18 expression on Huh7.5 cells treated with LPS or TNF was quantified by using flow cytometry as previously described (28). Briefly, cells were fixed, permeabilized, and stained with mouse anti-human USP18 polyclonal antibody (Abnova) or a relevant isotype-matched control antibody. Staining was detected with a secondary goat anti-mouse IgG labeled with fluorescein isothiocyanate (FITC; Santa Cruz Biotechnology). The cells were acquired using a FACSCalibur cytometer (BD Biosciences, San Jose, CA). A minimum of 10,000 events were collected. The resulting data were analyzed using FlowJo software (Tree Star, Inc.). The experiments were repeated four times.

### siRNA targeting murine USP18 and UBE1L.

USP18 small interfering RNA (siRNA) is a pool of three target-specific 19- to 25-nucleotide (nt) siRNAs designed to knock down USP18 gene expression that was obtained from Santa Cruz Biotechnology. The UBE1L siRNA used was a pool of three to five target-specific 19- to 25-nt siRNAs designed to knockdown mouse UBE1L gene expression and was obtained from Santa Cruz. USP18 and UBE1L siRNA was transfected into 5 × 10^5^ primary murine hepatocytes according to the manufacturer's instructions using the Santa Cruz siRNA reagent system sc-45064 (Santa Cruz) and as previously described (14).

### Inhibition of inflammatory signaling in primary mouse hepatocytes.

To inhibit LPS- or TNF-α-stimulated NF-κB activation, primary mouse hepatocytes were incubated with the following inhibitors for 30 min prior to 6 h of stimulation with LPS or TNF-α: P6062, a protein kinase A inhibitor (29); NSC33994, a Jak2 inhibitor (30); P3115, a protein kinase C inhibitor (31); PD098059, a mitogen-activated protein kinase kinase inhibitor (32); silibinin, an inhibitor of IKKα (33); and wortmannin, a phosphatidylinositide 3-kinase inhibitor (34). The concentrations, sources, and main targets of these inhibitors are described in Table 1. After LPS or TNF-α stimulation, hepatocytes were harvested and USP18 and IL-1β (a surrogate of NF-κB activation) mRNA expression was evaluated as described below.

**TABLE 1:** Inhibition of inflammatory signaling impairs TNF-α- and LPS-induced USP18 expression^*a*^

Inhibitor	Target	Concn (μM)	Avg decrease (%) in:	
			TNF-α-induced USP18 expression	LPS-induced USP18 expression
P6062	Protein kinase A	15	82	62
NSC33994	Jak2	25	81	87
P3115	Protein kinase C	75	86	34
PD 098059	Mitogen-activated protein kinase inhibitor	20	83	36
Silibinin	IKKα	80	78	83
Wortmannin	Phosphatidylinositol 3-kinase	10	84	9

a All inhibitors were obtained from Sigma.

### RNA isolation and quantitative real-time PCR analysis.

Huh7.5 cells and primary murine hepatocytes were treated with IFN-α (100 U/ml), TNF-α (20 ng/ml), LPS (100 ng/ml), IL-6 (100 ng/ml), or IL-10 (10 ng/ml) over a 24-h time course, and USP18 expression was determined by quantitative PCR (qPCR) as previously described (8, 9). Briefly, total RNA was prepared from cells using the TRIzol reagent (Invitrogen) according to the manufacturer's instructions. The reverse transcription reactions of the extracted RNA were performed with a first-strand cDNA synthesis kit (Amersham Pharmacia Biotech) according to the manufacturer's directions. First, 1 μg of extracted RNA was added in a total volume of 15 μl of combined cDNA reaction reagents with random hexamer oligonucleotides as the first-strand primer in a 1.5-ml reaction tube. Samples were heated to 65°C for 10 min, chilled on ice for 5 min, and incubated at 37°C for 1 h, followed by a 10-min incubation at 80°C. The specific primers for all of the detected genes for the PCRs were based on GenBank-published sequences: Mus musculus ubiquitin-specific peptidase 18 (USP18; NM_011909) forward primer (5′-TACAGCAGAGAGCAGCAGGA) and reverse primer (5′-CACATGTCGGAGCTTGCTAA); Mus musculus myxovirus (influenza virus) resistance 1 (Mx1; NR_003520) forward primer (5′-TCTGAGGAGAGCCAGACAAT-3′) and reverse primer (5′-ACTCTGGTCCCCAATGACAG); mouse hypoxanthine guanine phosphoribosyltransferase (HPRT; NM_013556) forward primer (5′-TCAGTCAACGGGGGACATAAA) and reverse primer (5′-GGGGCTGTACTGCTTAACCAG); Mus musculus IL-1β (NM_008361) forward primer (5′-GAAATGCCACCTTTTGACAGTG) and reverse primer (5′-TGGATGCTCTCATCAGGACAG); Homo sapiens IFN-α2b (IFNA2b; AY255838) forward primer (5′-GCTTGGGATGAGACCCTCCTA) and reverse primer (5′-CCCACCCCCTGTATCACAC); Homo sapiens IFN-γ (IFNG; NM_000416) forward primer (5′-TCGGTAACTGACTTGAATGTCCA) and reverse primer (5′-TCGCTTCCCTGTTTTAGCTGC); Homo sapiens actin, beta-like 2 (ACTBL2; NM_001017992) forward primer (5′-GTCTGCCTTGGTAGTGGATAATG) and reverse primer (5′-TCGAGGACGCCCTATCATGG); Homo sapiens ubiquitin specific peptidase 18 (USP18; NM_017414) forward primer (5′-AGGAGAAGCGTCCCTTTCCA) and reverse primer (5′-TGGTCCTTAATCAGGTTCCAGAG); and Homo sapiens myxovirus (influenza virus) resistance-1 (MX1; NM_001144925) forward primer (5′-GGTGGTCCCCAGTAATGTGG) and reverse primer (5′-CGTCAAGATTCCGATGGTCCT).

Quantitative real-time PCR was performed on an ABI Prism 7900HT machine (Applied Biosystems, Foster City, CA) with SYBR green real-time PCR master mix (Applied Biosystems) according to the directions provided by the manufacturer. All of the RNA samples and controls were assayed in duplicate. Real-time PCR conditions were as follows: 10 min at 95°C, followed by 45 cycles of 15 s at 95°C and 15 s at 60°C monitor fluorescence in SYBR channel during a 60°C annealing/extension step. The results were analyzed by using Applied Biosystems SDS2.2 software (Applied Biosystems).

### Experimental hepatic ischemia/reperfusion injury model.

Hepatic ischemia/reperfusion injury (HIRI) was simulated as before (35). Partial (70%) hepatic ischemia was induced for 60 min in mice, after which the surgical clamps were removed. Control (Sham) animals underwent anesthesia and laparotomy alone. Animals were euthanized 6 or 24 h after ischemia/reperfusion, the affected liver segments were removed, and target gene expression was determined by qPCR in whole liver tissue. LCMV strain WE was propagated in L929 cells (ATCC CCL-1) as previously described (36). In additional experiments, after 60 min HIRI or sham laparotomy, mice were infected with 2 × 10^6^ PFU of LCMV WE by intravenous tail vein injection. The ischemic liver lobes were harvested at day 7 postinfection. Snap-frozen liver tissue samples were homogenized in α-MEM (Multicell, USA) supplemented with 5% FBS, l-glutamine, and 100 U of penicillin/ml plus 100 μg of streptomycin/ml using the TissueLyser LT system (Qiagen, Netherlands). Viral titers were examined on MC57 cells (ATCC CRL-2295) using a focus-forming assay as previously described (37).

### Statistical analysis.

**FIG 1:**
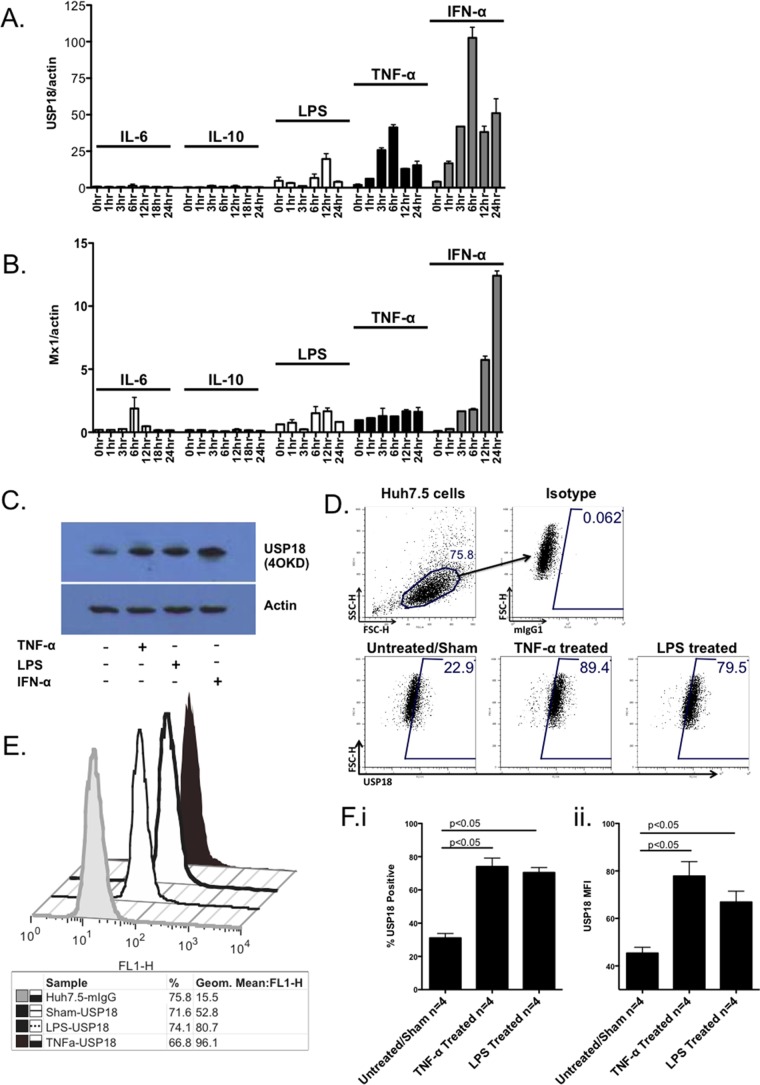
USP18 expression is upregulated by TNF-α and LPS. Huh7.5 cells were treated with LPS (100 ng/ml), TNF-α (20 ng/ml), IL-6 (100 ng/ml), IL-10 (10 ng/ml), or IFN-α (100 U/ml) for the times indicated, and ISG induction was measured by several approaches. (A and B) After stimulation with LPS, TNF-α, IL-6, IL-10, and IFN-α over a 24-h time course, RNA was harvested, and real-time PCR was performed. USP18 mRNA (A) and Mx1 mRNA (B) expression was determined and normalized to the expression of actin. Each data point was pooled from three samples. Error bars represent the standard errors of the mean (SEM) for duplicate PCRs. (C) Following stimulation with LPS, TNF-α and IFN-α for 12 h, Huh7.5 cells were lysed and USP18 protein expression was determined by Western blotting. (D and F) After stimulation with LPS, TNF-α, or SHAM for 24 h, Huh7.5 cells were fixed, permeabilized, and stained with an anti-USP18 monoclonal antibody, followed by a secondary anti-mouse FITC-labeled antibody. Representative flow plots (D) and representative histograms (E) show the degree of USP18 induction in Huh7.5 cells after LPS or TNF-α treatment. (F) Summary data showing the percentage of USP18-positive Huh7.5 cells (i) and the USP18 mean fluorescence intensity (MFI) (ii) in Huh7.5 cells after LPS or TNF-α treatment. The means ± the SEM from four independent replicates are plotted. For panels A to C, an unstimulated control (Sham) was included to identify background USP18 expression. A *P* value of <0.05 was considered significant.

All data were analyzed using Prism version 5.0 software (GraphPad Software, San Diego, CA). Statistically significant differences in Fig. 1E were calculated by using a two-tailed Student t test (Prism software). The impact of HIRI on LCMV replication (see Fig. 6A and B) was evaluated by one-way analysis of variance with the Tukey's post hoc test.

## RESULTS

### USP18 is induced in hepatocytes by LPS and TNF-α but not by IL-6 and IL-10.

We have previously shown that USP18 can modulate the type 1 IFN response (14). We sought to determine whether inflammatory stimuli could increase USP18 expression in hepatocytes. Liver tissue inflammation is mediated by both pro- and anti-inflammatory stimuli, acting through diverse pathways. We therefore compared the effects of three proinflammatory stimuli (TNF-α, LPS, and IL-6) and one anti-inflammatory stimulus (IL-10), all four signaling through independent receptors and signaling cascades (38, 39). In a 24-h time course experiment, USP18 mRNA expression was measured after stimulation with LPS (100 ng/ml), TNF-α (20 ng/ml), IL-6 (100 ng/ml), or IL-10 (10 ng/ml). USP18 mRNA expression was induced by TNF-α and LPS but not by IL-6 or IL-10 (Fig. 1A). Meanwhile, neither TNF-α nor LPS treatments had a marked effect on Mx1 mRNA expression (Fig. 1B), indicating that the effects we observe are not due to a generalized upregulation of ISGs or to type 1 IFN signaling. TNF-α and LPS treatment also led to augmented USP18 protein expression by Western blotting (Fig. 1C) and by intracellular staining (Fig. 1D to F). Treatment of Huh7.5 cells with TNF-α or LPS induced expression of USP18 in 74.1% ± 8.9% and 70.5% ± 5.2%, respectively, compared to untreated controls in which USP18 expression was 31.0% ± 4.7% (Fig. 1Fi). USP18 induction was also demonstrated with a significant shift in the mean fluorescence intensity of USP18 staining after TNF-α or LPS simulations (Fig. 1Fii). In these assays, the degree of protein expression did not correlate completely with mRNA expression, a finding that is consistent with many genes in the liver (40) in that the degree of mRNA induction was higher than the observed USP18 protein expression. However, the functional effects we observed in terms of IFN-α responses were robust.

### TNF-α and LPS block IFN-α signaling in Huh7.5 hepatoma cells.

**FIG 2:**
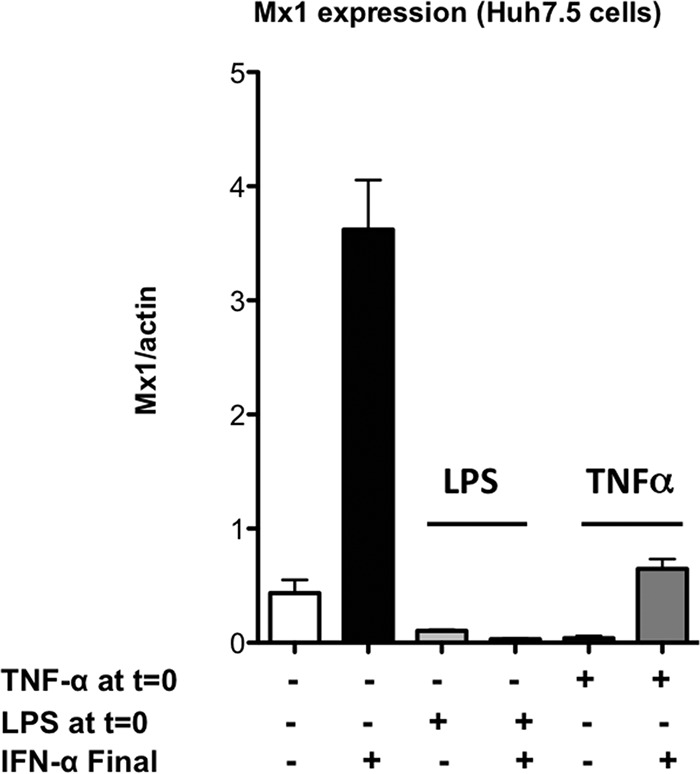
TNF-α and LPS block IFN-α signaling in Huh7.5 hepatoma cells. Human Huh7.5 hepatoma cells were cultured in the presence or absence of LPS (100 ng/ml) or TNF-α (20 ng/ml) for 24 h and either left untreated or treated with IFN-α (100 U/ml) for 6 h (IFN-α Final). The cells were harvested, and qPCR was performed for Mx1 as an index of IFN-α signaling. The data were generated from pooled triplicate experiments analyzed in duplicate. Error bars represent the SEM for duplicate PCRs.

To determine whether inflammatory stimuli such as TNF-α and LPS can interfere with type 1 IFN signaling, we sought to determine whether pretreatment of hepatoma cells with TNF-α or LPS blocked IFN-α signaling. Huh7.5 cells were treated with TNF-α (20 ng/ml) or LPS (100 ng/ml) for 24 h. The cells were subsequently treated with IFN-α for 6 h. We chose 6 h to measure Mx1 expression based on previous descriptions of IFN-stimulated Mx1 induction in the literature (41) and based on our observations with primary mouse hepatocytes. Control cells were left untreated, followed by a 6-h IFN-α treatment at 24 h. After the 6-h IFN-α treatment, the cells were harvested, and Mx1 expression was measured by qPCR as a measure of IFN-inducible gene expression. As expected, a 6-h exposure to IFN-α strongly induced Mx1 expression, whereas a 24-h exposure to TNF-α and LPS did not (Fig. 2, columns 2, 3, and 5). Both TNF-α and LPS pretreatment inhibited the effect of 6 h of exposure to IFN-α (Fig. 2, columns 2, 4, and 6), indicating that IFN-α signaling was impaired in the presence of these inflammatory stimuli.

### USP18 knockdown but not UBE1L knockdown reverses the refractory state induced by TNF-α and LPS in both Huh7.5 cells and primary hepatocytes.

**FIG 3:**
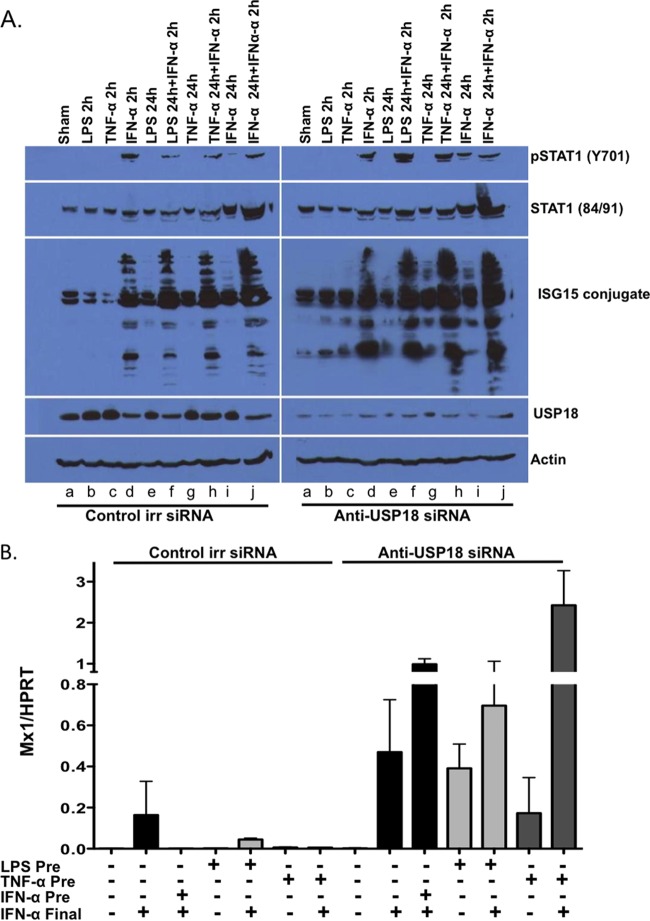
USP18 knockdown restores ISG induction after LPS and TNF-α stimulation. To look at the role of USP18 in the IFN-α refractory state induced by inflammatory stimuli, we examined the induction of STAT-1 phosphorylation in Huh7.5 cells with or without USP18 knockdown (A) and the expression of ISG mRNA (Mx1) in primary mouse hepatocytes and the ability of USP18 knockdown to restore ISG induction after LPS and TNF-α stimulation (B). (A) Huh7.5 cells were transfected with anti-USP18 siRNA or control irrelevant siRNA. Huh7.5 cells were then pretreated with LPS (100 ng/ml), TNF-α (20 ng/ml), or IFN-α (100 U/ml) or left untreated for 24 h and then exposed to IFN-α or left untreated for an additional 2 h. As controls, Huh7.5 cells were treated with LPS, TNF-α, and IFN-α for 2 h only. The expression of USP18, pSTAT1, STAT1, and ISG15 conjugates and actin proteins was measured by Western blotting. (B) Primary murine hepatocytes from USP18^+/+^ mice were isolated, transfected with anti-USP18 siRNA or control irrelevant siRNA, and pretreated with IFN-α (100 IU/ml), LPS (100 ng/ml), or TNF-α (20 ng/ml) for 24 h (IFN-α Pre, LPS Pre, or TNF-α Pre, respectively). After being washed, the cells were cultured in the presence or absence of IFN-α for 6 h (IFN-α Final). Mx1 ISG mRNA expression was measured by qPCR and normalized to the expression of the HPRT housekeeping gene. The data were generated from pooled triplicate experiments analyzed in duplicate. Error bars represent the SEM for duplicate PCRs.

Having shown that certain inflammatory stimuli both increase hepatocyte USP18 and blunt IFN-α signaling, we next sought to determine whether the blunting of IFN-α signaling is mediated via increased USP18. We addressed this question by selective knockdown of USP18 mRNA. Thus, Huh7.5 cells transfected with USP18 siRNA or control siRNA were treated with LPS (100 ng/ml), TNF-α (20 ng/ml), and/or IFN-α (100 IU/ml) for 2 h or pretreated with LPS (100 ng/ml) or TNF-α (20 ng/ml) for 24 h and then either left untreated or treated with IFN-α (100 IU/ml) for 2 h (a 2-h time point was selected for optimal expression of pSTAT1 in Huh7.5 cells). The expression of pSTAT1, STAT1, and ISG15 conjugates (as a marker of USP18 knockdown), USP18, and actin was detected by Western blotting. As seen in Fig. 3A, USP18 expression was largely knocked down in cells transfected with USP18 siRNA. IFN-α-induced phosphorylation of STAT-1 was inhibited by pretreatment (for 24 h) with TNF-α or LPS (Fig. 3A, siRNA control lanes f and h versus lane d), but this effect is reversed by knockdown of USP18 (Fig. 3A, siRNA USP18, lanes f and h versus lane d). Of note, USP18 knockdown did not increase pSTAT1 in response to IFN-α before 24 h; these findings are consistent with previous observations (12). We next wanted to confirm whether our findings from hepatoma cells would hold true in primary mouse hepatocytes. With this in mind, primary murine hepatocytes from USP18^+/+^ mice were isolated, transfected with USP18 siRNA or control siRNA, and then exposed to IFN-α (100 IU/ml), LPS (100 ng/ml), or TNF-α (20 ng/ml) for 24 h. After a washing step, the cells were treated with IFN-α for an additional 6 h. Mx1 ISG mRNA expression was measured by qPCR (normalized to expression of the HPRT housekeeping gene) as an index of downstream IFN-α effect. IFN-α, LPS, and TNF-α treatment blocked the effect of the final dose of IFN-α (Fig. 3B, columns 2, 3, 5, and 7). However, knockdown of USP18 reversed this effect and augmented the effect of IFN-α (Fig. 3B, columns 9, 10, 12, and 14). The data from this experiment are consistent with those obtained with human Huh7.5 cells (data not shown). Thus, the ability of TNF-α and LPS to block IFN signaling is seen in both primary and immortalized hepatocytes.

**FIG 4:**
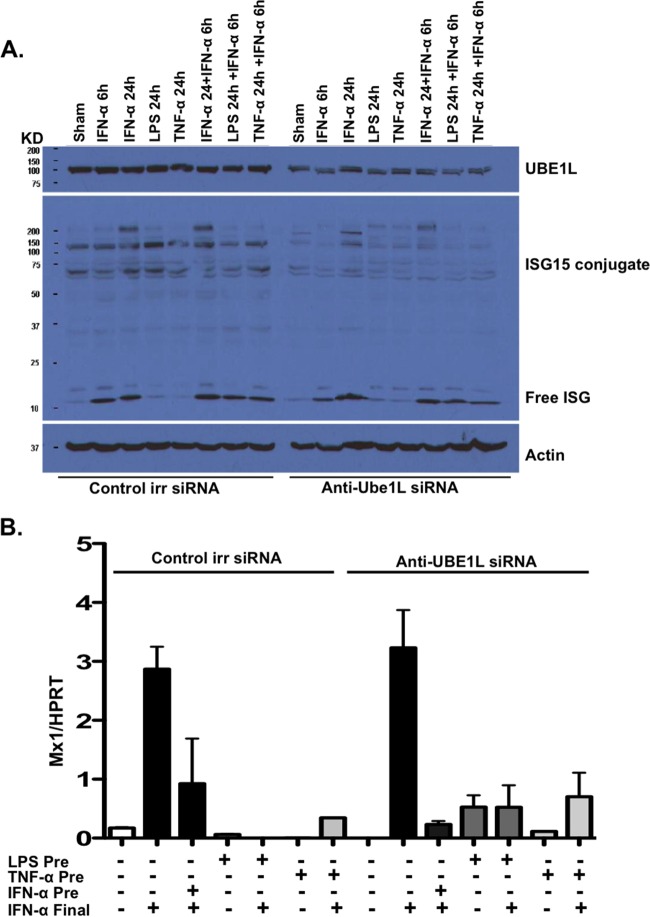
ISG15 deubiquitination does not play a role in USP18-mediated IFN-α refractoriness. To examine the role of ISG15 deubiquitination in the IFN-α refractory state induced by inflammatory stimuli, we used UBE1L knockdown and then measured TNF-α- and LPS-induced IFN refractoriness. (A) USP18^+/+^ murine hepatocytes were transfected with anti-UBE1L siRNA or control irrelevant siRNA were stimulated with IFN-α (100 IU/ml), LPS (100 ng/ml), or TNF-α (20 ng/ml) for 24 h and then left untreated or were treated with an additional dose of IFN-α for 6 h. Expression of free ISG, ISG15 conjugates, UBE1L, and actin proteins were measured by Western blotting. (B) USP18^+/+^ murine hepatocytes transfected with anti-UBE1L siRNA or control irrelevant siRNA were pretreated with IFN-α (100 IU/ml), LPS (100 ng/ml), or TNF-α (20 ng/ml) for 24 h (IFN-α Pre, LPS Pre, or TNF-α Pre, respectively) and then left untreated or treated with an additional dose of IFN-α for 6 h (IFN-α Final). Mx1 ISG mRNA expression was measured by qPCR and normalized to the expression of the HPRT housekeeping gene. The data were generated from pooled triplicate experiments analyzed in duplicate. Error bars represent the SEM for duplicate PCRs.

As noted earlier, USP18 has dual roles: it both strips ISG15 from its target proteins and impairs type 1 IFN signaling independent of its protease activity (15). To determine whether the USP18-dependent blunting of hepatocyte IFN signaling was due to its ability to strip ISG15 from its conjugates, we blocked the process of ISGylation by knockdown of the ISG15 E1 enzyme, UBE1L. Murine hepatocyte UBE1L was knocked down via transfection with siRNA specific for UBE1L. USP18^+/+^ murine hepatocytes transfected with UBE1L siRNA or control siRNA were stimulated with IFN-α (100 IU/ml), LPS (100 ng/ml), or TNF-α (20 ng/ml) for 24 h and then treated with an additional dose of IFN-α for 6 h. Knockdown was measured in the hepatocytes transfected with UBE1L siRNA by Western blotting probing for UBE1L expression and ISG15 conjugate formation (Fig. 4A). Next, to examine the effect of this knockdown on ISG induction, wild-type murine hepatocytes transfected with UBE1L siRNA or control siRNA were stimulated with TNF-α (20 ng/ml) and LPS (100 ng/ml) for 24 h and then restimulated with IFN-α (100 IU/ml) for 6 h, at which time Mx1 ISG mRNA expression was measured by qPCR. As seen in Fig. 4A, UBE1L knockdown was achieved, resulting in a decrease in ISG15 conjugates. However, as seen in Fig. 4B, we observed that neither the presence nor the relative absence of ISG15 conjugation impacted the ability of TNF-α and LPS to block IFN-α signaling since there was no change in ISG expression after 6 h of IFN-α treatment when USP18^+/+^ hepatocytes transfected with anti-UBE1L siRNA were compared to hepatocytes transfected with irrelevant siRNA (Fig. 4B, siRNA control, columns 2, 5, and 7, and UBE1L siRNA, columns 9, 12, and 14). These results are consistent with there being no role for ISGylation (and, thus, for the ability of USP18 to strip ISG15 from its protein conjugates) in the ability of TNF-α and LPS to block type 1 IFN signaling in hepatocytes, and this finding is in agreement with previous data showing that USP18 blocks IFN signaling independent of its enzymatic activity (15).

### IFN-α, LPS, and TNF-α do not induce type 1 and 2 IFNs in primary murine hepatocytes but do induce USP18 expression.

**FIG 5:**
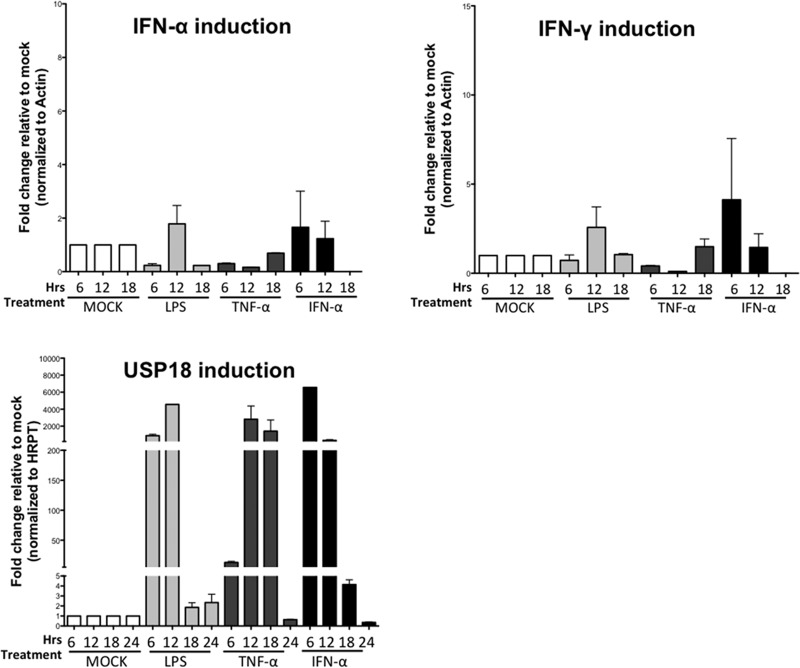
IFN-α, LPS, and TNF-α do not induce type 1/2 IFN in primary murine hepatocytes but do induce USP18. Primary murine hepatocytes were treated with IFN-α (100 IU/ml; black bars), LPS (100 ng/ml; light gray bars), TNF-α (20 ng/ml; dark gray bars), or mock treated (white bars) for the times indicated and then lysed and assessed for IFN-α, IFN-γ, and Mx1 expression by qPCR. ISG mRNA expression was normalized to expression of the HPRT and actin housekeeping genes prior to determining the fold increase versus mock-treated samples. The data were generated from pooled triplicate experiments analyzed in duplicate. Error bars represent the SEM for duplicate PCRs.

We next sought to determine whether TNF-α and LPS could induce USP18 expression in hepatocytes via the induction of IFN-α. As observed in Fig. 3A, STAT1 phosphorylation shows distinct activation profiles with IFN-α stimulation compared to TNF-α or LPS stimulation (Fig. 3A, siRNA control and siRNA USP18, lanes b and c compared to lane d). Although these data strongly suggest that the effect of LPS and TNF-α stimulation are a result of direct induction of ISGs and not secondary to the induction of IFN-α, we wanted to confirm whether LPS and TNF-α could induce type 1 or type 2 IFN (IFN-α or IFN-γ) in primary murine hepatocyte. Thus, primary murine hepatocytes were treated with IFN-α (100U/ml), LPS (100 ng/ml), or TNF-α (20 ng/ml) for 6, 12,18, or 24 h; they were then lysed and assessed for IFN-α, IFN-γ, and USP18 expression by qPCR. We observed that neither IFN-α, LPS, nor TNF-α induce much, if any, hepatocyte expression of IFN-α or IFN-γ, although the same doses induce strong expression of USP18 (Fig. 5). Thus, the induction of ISGs by LPS and TNF-α is very unlikely to reflect the induction of hepatocyte type 1 or type 2 IFN, which suggests that the observed USP18 induction is not due to type 1 or type 2 IFN secretion and autocrine stimulation of the IFN-α receptor.

### Experimental hepatic ischemia/reperfusion induces USP18 expression and enhances LCMV replication.

**FIG 6:**
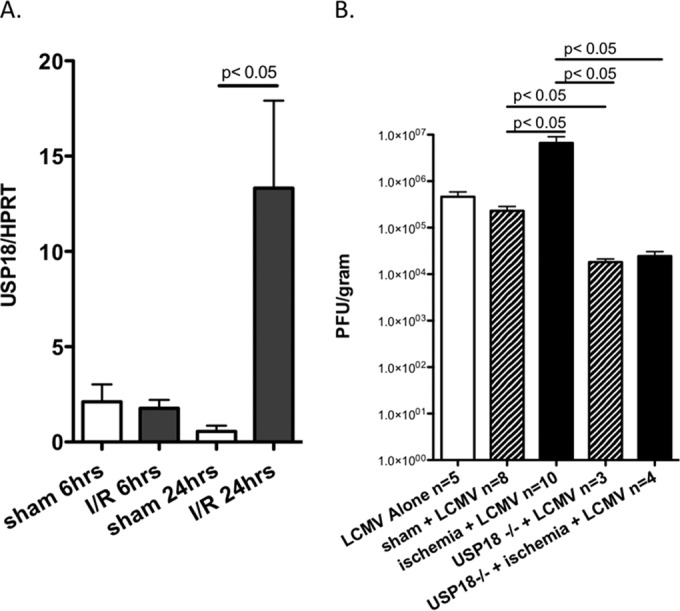
Experimental hepatic ischemia/reperfusion induces USP18 expression and enhances LCMV replication. To examine the *in vivo* effect of an inflammatory stimulus on USP18 expression and viral replication, a hepatic ischemia/reperfusion model was used. (A) Partial (70%) hepatic ischemia was induced for 60 min, after which the portal vascular clamp was removed. Control animals (Sham) underwent anesthesia and a laparotomy alone. Animals were euthanized 6 and 24 h after ischemia/reperfusion (I/R), and the affected liver segments were removed. USP18 mRNA expression was determined by qPCR in whole liver tissue and normalized to hypoxanthine-guanine phosphoribosyltransferase (HPRT) expression. Data are expressed as means ± the SEM with *n* = 3 to 4 mice/group. A *P* value of <0.05 was considered significant. (B) After 60 min of HIRI or sham laparotomy, mice were infected with 2 × 10^6^ PFU of LCMV WE. Liver lobes were harvested at day 7 postinfection. Viral titers were examined on MC57 cells using a focus-forming assay. Data are expressed as means ± the SEM with *n* = 3 to 10 mice/group. A P value of <0.05 was considered significant.

We then assessed whether tissue-wide hepatic inflammatory stress increases liver USP18 expression, as an *in vivo* confirmation of our *in vitro* findings. This was approached by inducing partial (70%) hepatic ischemia/reperfusion injury (HIRI) for 60 min in mice, euthanizing the animals at 6 or 24 h (time points relevant to our *in vitro* time course) and measuring induction of USP18 by qPCR. We chose HIRI as a very well-characterized inflammatory stress, known to be driven both by TNF-α and LPS (35). As seen in Fig. 6A, HIRI alone induced USP18 mRNA expression in whole livers by >10-fold that of untreated animals. Thus, *in vivo* liver inflammation leads to increased USP18 expression at the organ level.

After determining that HIRI induces USP18 expression, we next wanted to examine the impact of HIRI on viral control. Thus, we induced 70% HIRI for 60 min prior to LCMV WE infection of USP18^+/+^ and USP18^−/−^ mice. As seen in Fig. 6B, HIRI led to a significant increase in LCMV viral titers in USP18^+/+^ mice, an effect that was not observed in USP18^−/−^ mice.

### Inhibition of inflammatory signaling impairs LPS and TNF-α stimulated USP18 induction.

**FIG 7:**
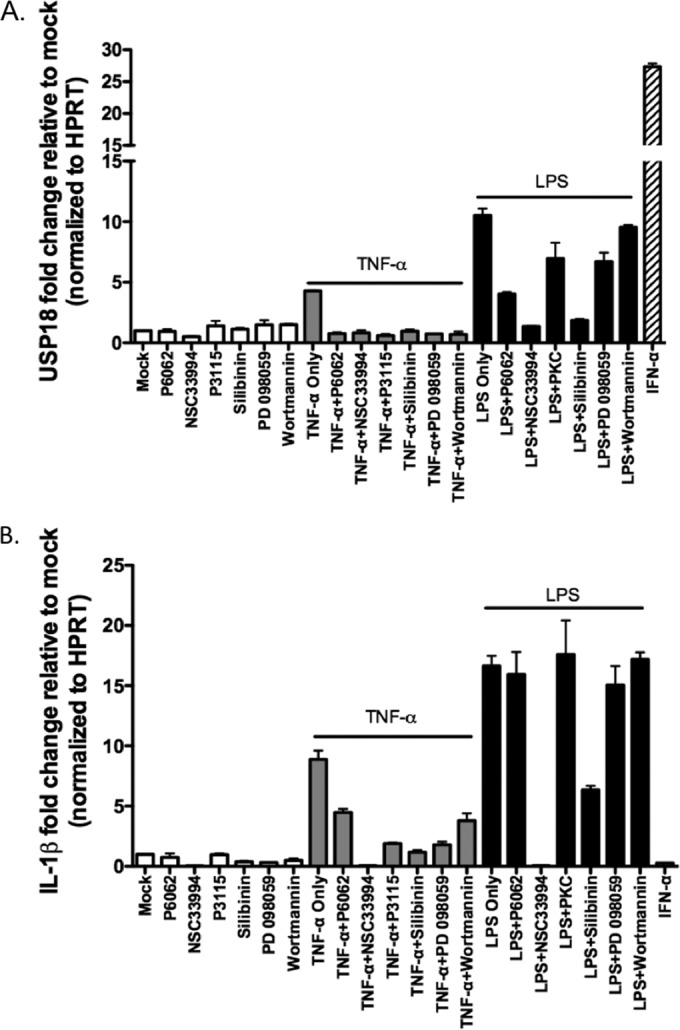
Inhibition of NF-κB activation impairs LPS- and TNF-α-stimulated USP18 induction. To investigate the link between LPS/TNF-α stimulation and USP18 induction, inhibitors of NF-κB activation were added to primary mouse hepatocytes for 30 min prior to stimulation with 20 ng of TNF-α/ml or 100 ng of LPS/ml for 6 h (the inhibitor names, targets, and concentrations used are given in Table 1). Inhibitor-treated cells were also left unstimulated as a control. As a control for USP18 induction, hepatocytes were stimulated with 100 U of IFN-α/ml for 6 h. USP18 (A) and IL-1β (B) mRNA expression levels were evaluated by PCR and normalized to the HPRT housekeeping gene expression prior to determining the fold increase versus mock-treated samples. The data were generated from pooled triplicate experiments analyzed in duplicate. Error bars represent the SEM for duplicate PCRs.

Having shown that LPS/TNF-α stimulation increases hepatocyte USP18 expression, we then sought to determine whether we could pharmacologically inhibit the induction of USP18 by LPS and TNF-α. We focused on small-molecule agents that have been linked to the NF-κB signaling pathway, because of the central role of this pathway in inflammatory activation in response to a large number of inflammatory mediators, including LPS and TNF-α (42). We were particularly interested in inhibitors, such as silibinin, that in addition have been linked to clinical suppression of hepatic viral production (in the case of silibinin and HCV) (43, 44). Thus, we preincubated primary mouse hepatocytes for 30 min with various inhibitors of LPS and TNF-α signaling and measured their impact on USP18 induction, while simultaneously measuring expression of proinflammatory cytokine IL-1β mRNA, since NF-κB is also known to promote IL-1β transcription (45, 46). As seen in Fig. 7A and as described in Table 1, TNF-α induced expression of USP18 was potently downregulated by all inhibitors tested, and this inhibition coincided with impaired IL-1β mRNA expression (Fig. 7B). Meanwhile, only two inhibitors potently (>80%) inhibited USP18 expression as well as IL-1β mRNA expression in response to LPS stimulation; NSC33994 (an inhibitor of Jak2) and silibinin (an inhibitor of IKKα) (Fig. 7A and B and Table 1). These data suggest that the induction of USP18 by TNF-α and LPS, and possibly other inflammatory stimuli, is promoted by NF-κΒ signaling and that hepatocyte USP18 expression in particular—compared to IL-1β—may be an attractive target for pharmacologic manipulation in the setting of liver inflammation.

## DISCUSSION

In this study we examined the role of various inflammatory stimuli in the induction of USP18 and the downstream establishment of an IFN-α refractory state. We used multiple methods to show that certain inflammatory stimuli, including LPS and TNF-α are able to induce the expression of USP18, which results in downstream downregulation of IFN-α-induced ISG expression. The role of USP18 in the IFN-α refractory state has been previously demonstrated by human and mouse USP18 knockdown studies (12, 14–16). Other inflammatory stimuli, including IL-10 and IL-6, did not induce USP18 or impair expression of ISGs, including USP18. *In vivo*, hepatic inflammatory stress (ischemia/reperfusion injury) led to increased hepatic USP18 gene expression that was associated with poor control of LCMV infection. Thus, the hepatic inflammatory milieu, contributed to by individual inflammatory cytokines and stimuli, modulates USP18 expression. These results demonstrate one mechanism by which liver inflammation directly impacts the hepatocellular innate immune response.

USP18 is known to be induced by multiple inflammatory stimuli in macrophages (18) and lymphocytes (47). Our findings in hepatocytes are consistent with work done by other groups in immune cells. For example, LPS treatment of a murine macrophage cell line upregulates USP18 in an IRF3-dependent manner (18). The cytokine specificity of our results is also consistent with finding that IL-6 alone is not able to induce USP18 in murine T cells (47). Only when T cells were treated with IL-6 and another proinflammatory cytokine, such as transforming growth factor β, IL-1β, and IL-23, was USP18 expression induced (47). These data point out that USP18 can be induced by inflammatory stimuli in multiple cell types in the absence of IFN. USP18 is therefore well positioned to act as the mediator of “cross talk” between innate immunity and inflammatory responses.

In the present study, we show that increased USP18 expression following exposure of hepatocytes by inflammatory stimuli blunts the IFN response. The binding of USP18 with the IFN-α receptor has been shown to inhibit the interaction of STAT-1 with the IFN-α receptor and thus block downstream IFN signaling (12, 15). Our data are consistent with this mechanism, since the blunting of hepatocyte IFN signaling after exposure to inflammatory stimuli is independent of USP18-mediated removal of ISG15 from its target proteins. The degree of TNF-α and LPS-induced IFN-α refractoriness was not changed by UBE1L knockdown, although ISGylation was considerably reduced. However, other mechanisms may also be at play. Recent work has demonstrated that USP18 has the ability to deubiquitinate the transforming growth factor-activated kinase 1 (TAK1) complexes required for NF-κB activation in T cells and that overexpression of USP18 leads to decreased nuclear activation and impaired formation of TAK1 complexes (47). USP18-mediated NF-κB inhibition may be of importance not only for T cell adaptive immunity but also for liver inflammation. The role of TAK1 in innate and adaptive immunity has been previously demonstrated (48), and studies have also shown that TAK1 deletion interferes with hepatocyte homeostasis and leads to hepatic injury (49). Thus, increased hepatocyte USP18 in response to inflammatory stimuli may constitute a negative-feedback cycle with relevance to multiple aspects of the liver's response to infection.

Our *in vivo* experiments demonstrated that inflammation resulting from ischemia/reperfusion injury induces USP18 expression, which coincides with diminished LCMV control in mouse livers. The general finding that liver inflammation promotes LCMV production is in agreement with work showing that polymicrobial sepsis, characterized by high TNF-α and inflammation, leads to increased susceptibility to LCMV infection (50). In our present study, hepatic ischemia/reperfusion injury did not enhance LCMV production in USP18 knockout mice. These data are intended as proof-of-concept but do suggest that the role of USP18 in hepatic viral infection and inflammation deserves further investigation.

Our *in vitro* and in vivo findings suggest a new model for how inflammation alters hepatic innate immune responses. In this model, liver inflammation leads to increased hepatocyte USP18, which in turn makes the liver more susceptible to infections targeting the hepatocyte, such as HCV. This mechanism may help to explain the clinical observation that HCV infection of a transplanted, HCV-naive liver graft (that has gone through an ischemia/reperfusion cycle) is more aggressive than the original infection and that the severity of the reinfection correlates with the severity of the ischemia/reperfusion injury suffered during the transplant (51, 52). However, we have also found that USP18 is necessary but not sufficient on its own to induce an IFN-α refractory state (53). With this in mind, we hypothesize that the innate immune response and its ability to control IFN-sensitive viruses will depend on cumulative effect of multiple intrahepatic signals, including the induction of USP18.

If these results are to be translated into a clinical setting, then one approach is to target USP18 induction pharmacologically. Using multiple inhibitors of TNF-α/LPS signaling, we found that two inhibitors, silibinin (an inhibitor of IKKα) and NSC33994 (a Jak2 inhibitor) possess the ability to inhibit TNF-α and LPS-induced USP18 expression and proinflammatory effects. These findings raise the possibility of pharmacologically targeting USP18 expression during inflammatory events to prevent the establishment of an IFN-α refractory state. Previously, inhibition of Jak2 signaling led to a protection of mouse livers from ischemic insult (54). As well, silibinin has been shown to reduce of HCV liver graft reinfection (43) and enhance HCV clearance in IFN-α nonresponders (55) via multiple mechanisms, including by direct inhibition of HCV NS5B RNA polymerase (44). USP18 modulation may be one mechanism by which silibinin exerts its anti-HCV effects.

The present study focuses on a relatively small number of inflammatory stimuli; while TNF-α and LPS are important to a large number of liver diseases, they are far from being the only drivers of the hepatic inflammatory response and tissue levels of USP18 are likely influenced by other stimuli as well. For example, we previously demonstrated that increased USP18 expression in the liver is predictive of patients with chronic HCV infection who will not respond to IFN-based anti-HCV therapy (8). Although we found that TNF-α hepatic mRNA expression was increased in treatment nonresponders, it was relatively more increased in treatment responders (9). We suspect that whereas increased TNF-α does contribute to USP18 expression, there are other stimuli, for example, LPS and perhaps the recently described interferon-lambda 4 (56), that also modulate hepatic USP18 expression. We propose that the ultimate USP18 expression is due to the liver's coordinate response to multiple stimuli. The finding that hepatic USP18 expression is modulated by inflammatory stimuli is a new paradigm for the interaction of the liver inflammatory microenvironment and viral infection.

Taken together, these data may suggest that USP18 represents a good target for intervention in numerous inflammatory states and in the clinical setting of HCV-related liver transplantation. HIRI-induced upregulation of USP18 could lead to worsened outcomes posttransplant, including a quicker, more aggressive reinfection of the new organ with higher HCV RNA titers. We suggest that the finding that IFN responses and USP18 expression are tightly linked to the hepatic inflammatory response helps to explain the finding that TNF-α antibody treatment improves treatment outcomes of chronic HCV with treatment combinations, including IFN-α (5).
